# Supplementary material for: Shared regulation and functional relevance of local gene co-expression revealed by single cell analysis
Source: Commun Biol. 2022 Aug 26;5:876. doi: 10.1038/s42003-022-03831-w (PMC9418141; doi:10.1038/s42003-022-03831-w)
Supplement: Supplementary file 2 — Supplementary Information [file 42003_2022_3831_MOESM2_ESM.pdf]

Supplementary Information

Supplementary Table 1 Summary of single cell datasets used and COP results. Values in parenthesis refer to means across individuals for the Cuomo *et al.* 2021 dataset. Number of COPs refers to distinct gene pairs.

Dataset	Cell line	Technology	# individuals	# cells	# COPs
Cuomo *et al.* 2021	iPSC (undifferentiated)	Smart-seq2	87	7440 (85.5)	3877 (113.3)
Ma *et al.* 2020	LCL (GM12878)	SHARE-seq	1	26,589	2589

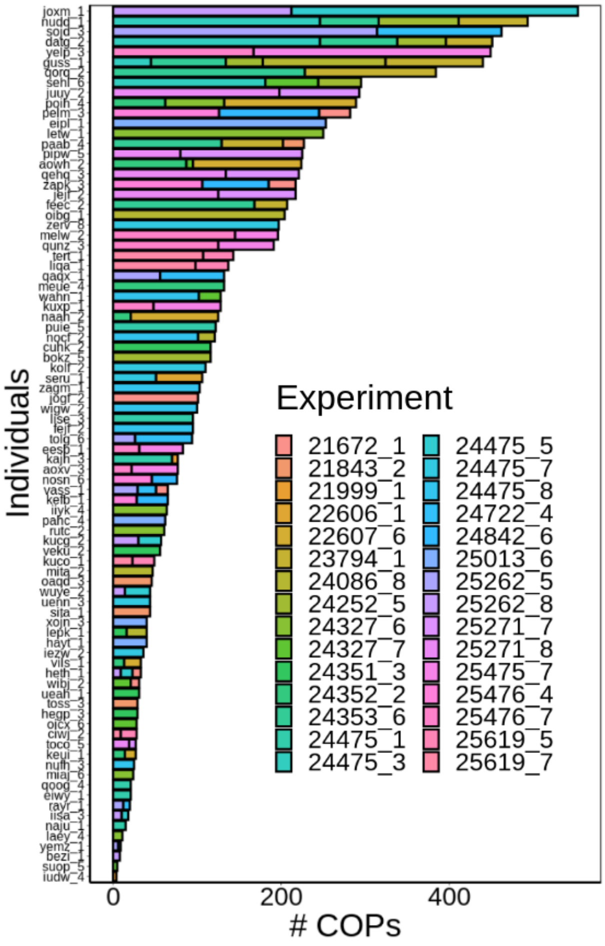

Supplementary Figure 1 Number of COPs identified per individual-experiment. The y-axis represents the individual and is sorted by total number of COPs, the experiment is color-coded. Multiple experiments are present for each individual. Note that the same COP can be identified in multiple experiments of the same individual.

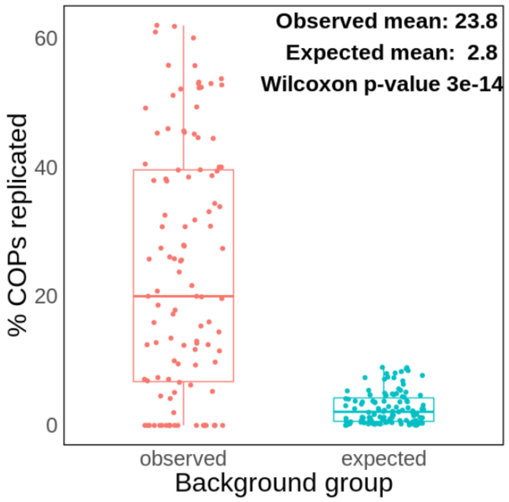

Supplementary Figure 2 Percentage of COPs replicated across multiple experiments of the same individual. The COPs of each individual-experiment are tested for replication across all other experiments of the same individual. Only 48 out of 87 individuals with more than one experiment were considered. The expected values were calculated by comparing the COPs to a sample with the same number of COPs as those in the other experiments, but drawn from experiments of different individuals. For instance, if calculating the replication rate of 10 COPs of a certain individual-experiment against 100 other COPs from other experiments of the same individual, these 100 COPs will instead be withdrawn from the pool of all COPs present across individuals. The length of the box corresponds to the interquartile range (IQR) with the centre line corresponding to the median, the upper and lower whiskers represent the largest or lowest value no further than 1.5 × IQR from the third and first quartile, respectively.

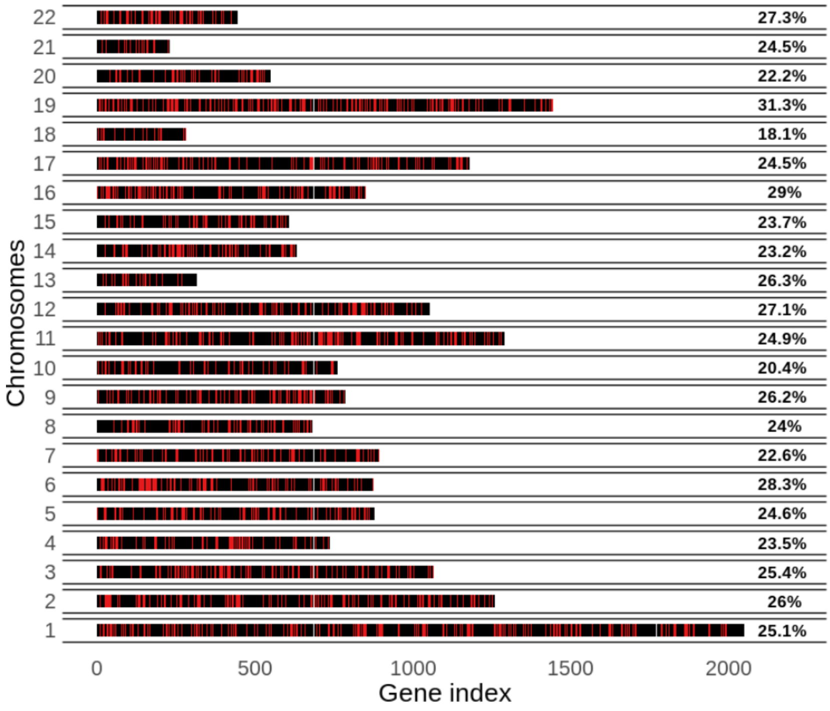

Supplementary Figure 3 Genes in COPs across chromosomes. Red represents a co-expressed gene, black a non-co-expressed gene. Genes are indexed by their genomic position in the chromosome. The percentages on the right side of each plot denote the percentage of genes that are co-expressed per chromosome.

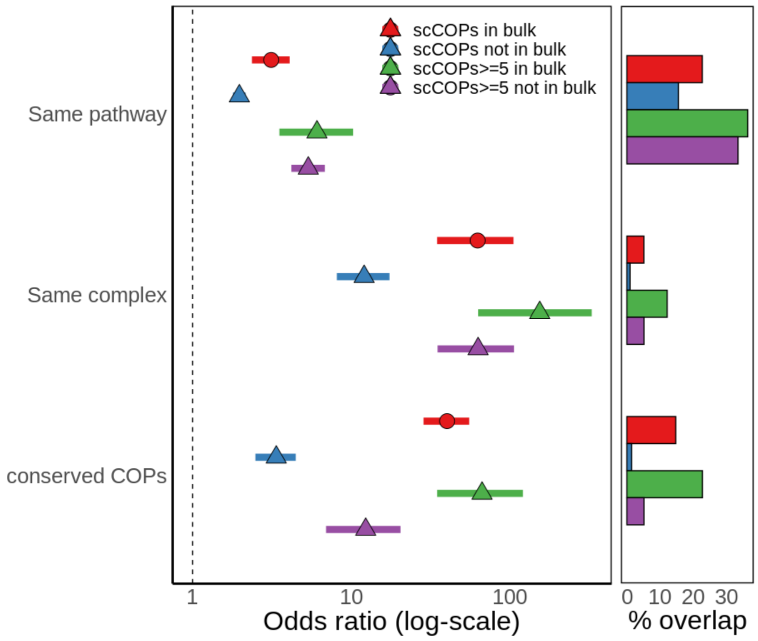

Supplementary Figure 4 scCOP enrichment for the same gene pathway, protein complex or GTEx conserved COPs separated by identification in bulk. "scCOPs>=5" are the subset of scCOPs found across 5 or more individuals. Odds ratios were calculated with one-sided Fisher's Exact tests and the 95% confidence interval is shown. X-axis is log-scaled, but values shown are before transformation. The right part of the plot denotes the percentage of COPs in each functional annotation.

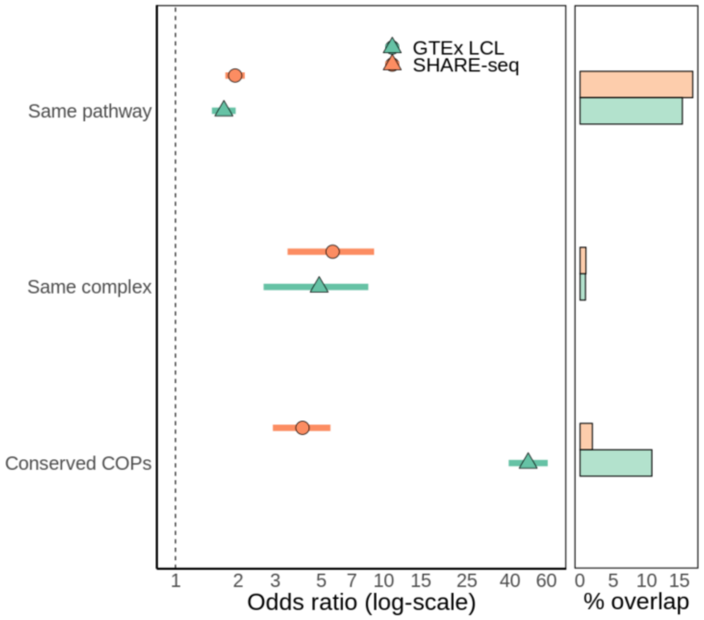

Supplementary Figure 5 COP enrichment for the same gene pathway, protein complex or GTEx conserved COPs for SHARE-seq LCL COPs. Odds ratios were calculated with one-sided Fisher's Exact tests and the 95% confidence interval is shown. X-axis is log-scaled, but values shown are before transformation. As a comparison, results for COPs identified for GTEx LCLs (Cells EBV-transformed lymphocytes) from Ribeiro *et al.* 2021[10] are also shown used. The right part of the plot denotes the percentage of COPs in each functional annotation.

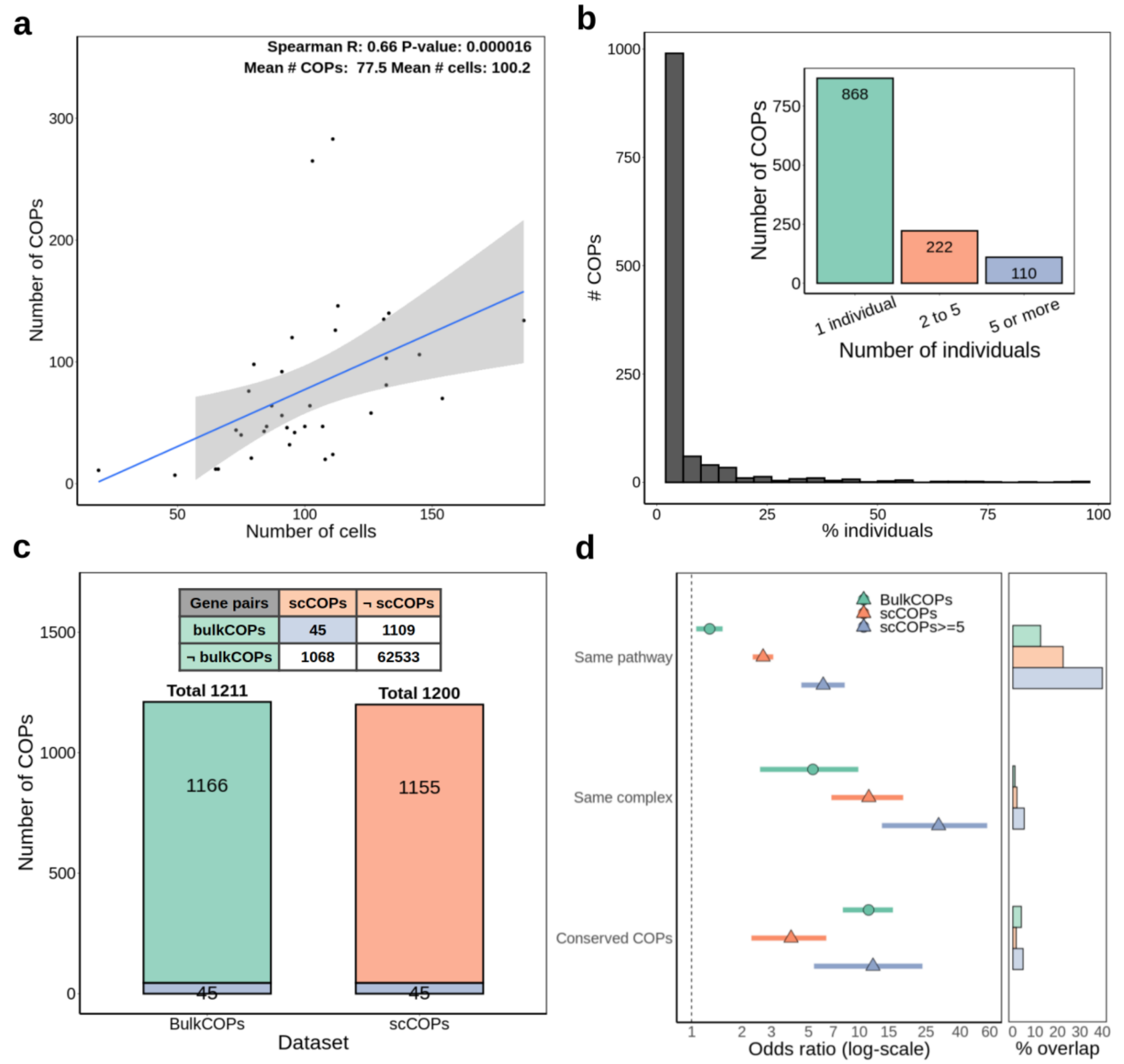

Supplementary Figure 6 Single cell COP and bulk COP discovery in the Sarkar et al. 2019 dataset (a) number of cells per individual and number of COPs mapped. Fit line corresponds to a linear regression model with 95% confidence intervals; **(b)** distribution of the percentage of individuals in which COPs are present. The inner plot counts how many COPs in 1, 2 to 5 (exclusive) and 5 or more individuals; **(c)** total number of COPs detected with bulk data (bulkCOPs) and single cell data (scCOPs, union across individuals). Numbers in green represent COPs found from both bulk and single cell data. The contingency table summarises the overlap between scCOPs and bulkCOPs considering the common background of gene pairs tested; **(d)** one-sided Fisher's exact test odds ratio enrichment (and 95% confidence interval) for the pair of genes in COPs to belong to the same gene pathway, protein complex or in the set of COPs conserved across GTEx tissues. "scCOPs>=5" are a subset of COPs that are found across 5 or more individuals. X-axis is log-scaled, but values shown are before transformation. The right part of the plot denotes the percentage of COPs in each functional annotation.

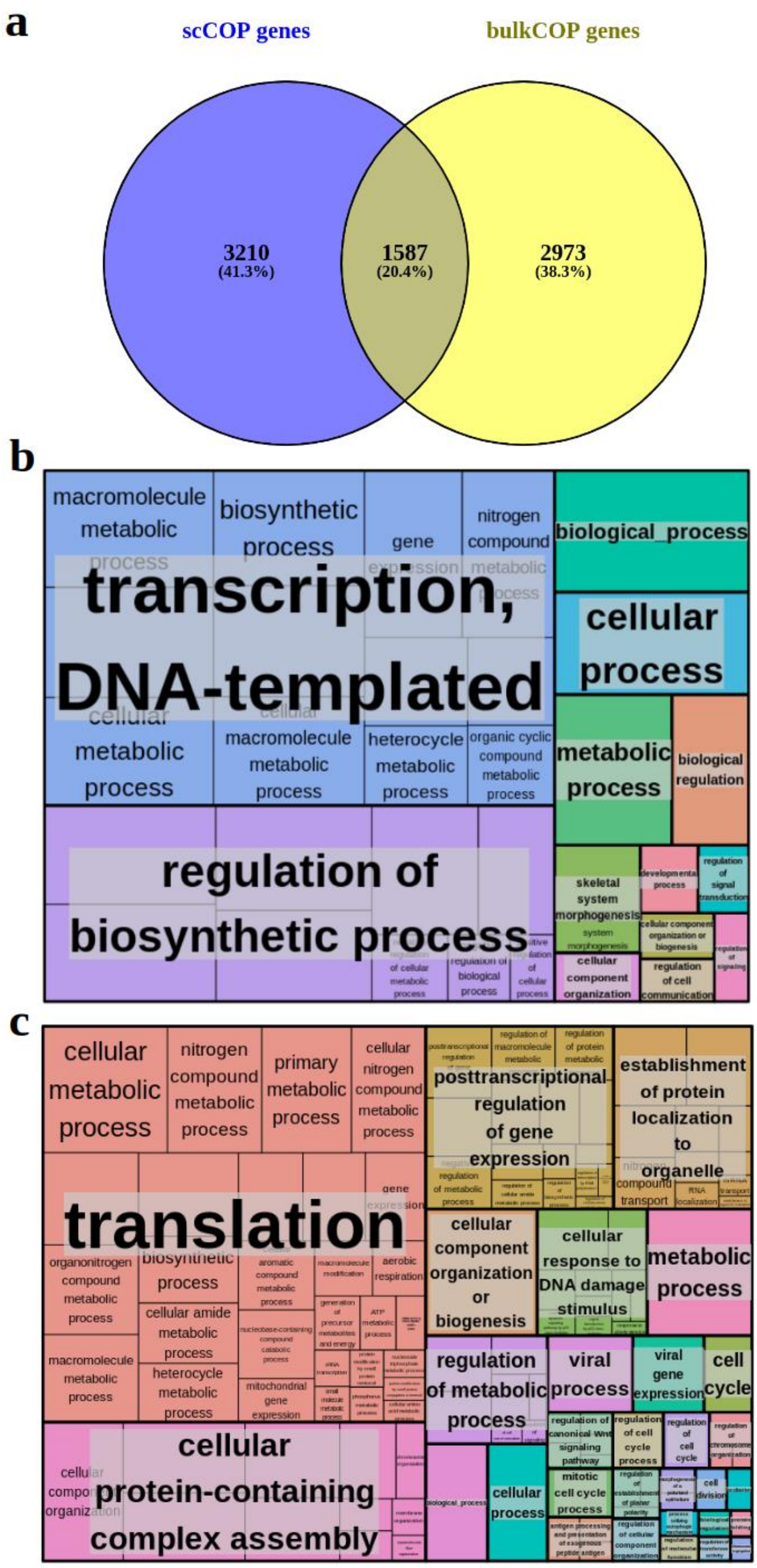

Supplementary Figure 7 Bulk and single cell COP overlap and annotation term enrichments. (a) overlap of COP genes between bulk and single cell COP discovery (Venny v2.1, https://bioinfogp.cnb.csic.es/tools/venny); **(b)** bulkCOP gene and **(c)** scCOP gene GO biological process enrichment (gprofiler), summarised with REVIGO (semantic similarity, see Methods).

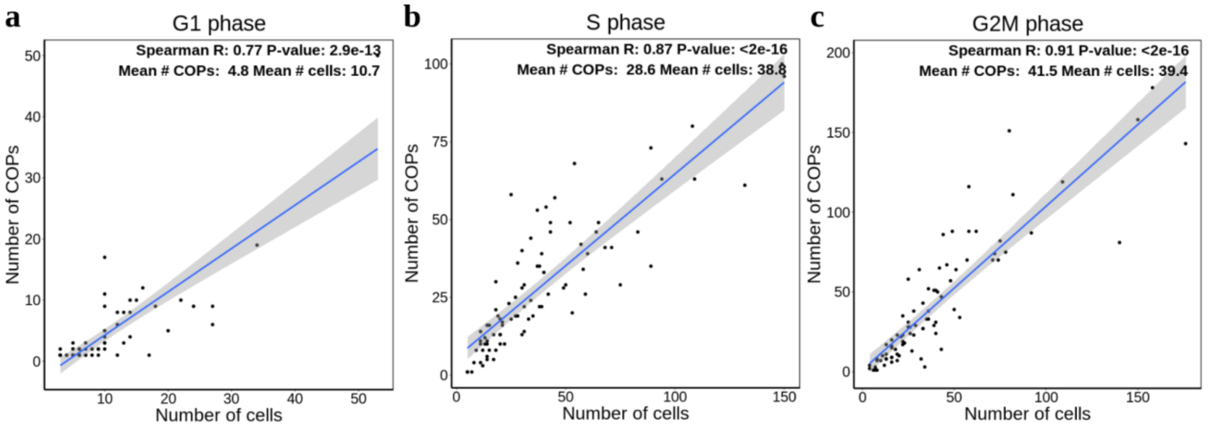

Supplementary Figure 8 Number of cells per individual and number of COPs mapped per cell cycle phase **(a)** G1 phase, **(b)** S phase and **(c)** G2M phase. Fit lines correspond to a linear regression model with 95% confidence intervals.

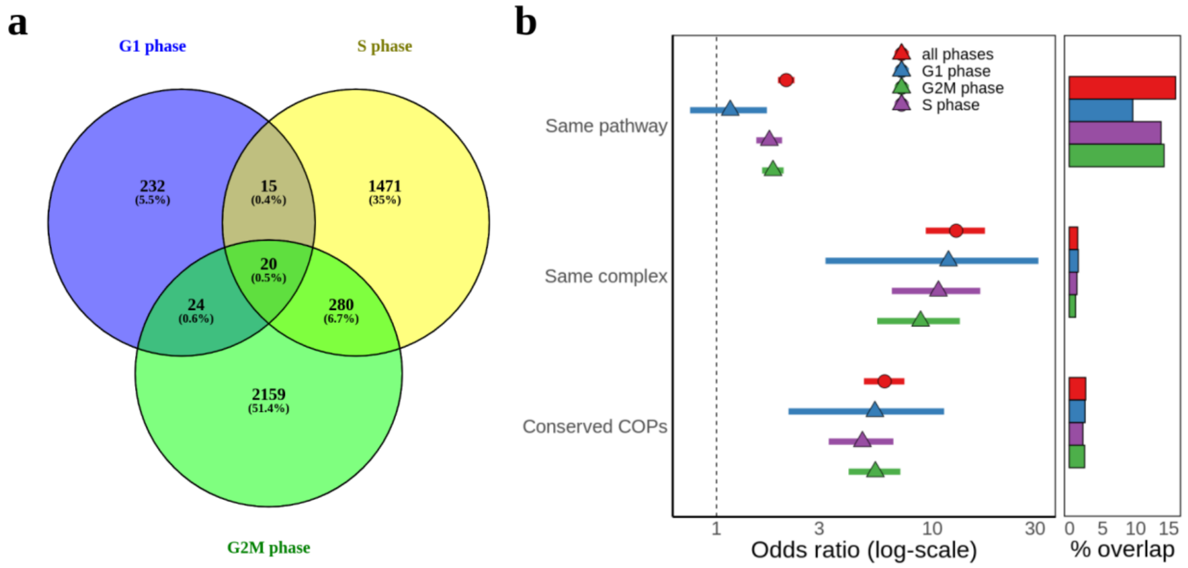

Supplementary Figure 9 Cell cycle phase COP overlap and functional enrichment (a) COP overlap between the G1, S and G2M phases (Venny v2.1, https://bioinfogp.cnb.csic.es/tools/venny); **(b)** COP enrichment for the same gene pathway, protein complex or GTEx conserved COPs for each cell cycle phase. ‘All phases’ represent the COP identification with all available cells. Odds ratios were calculated with one-sided Fisher’s Exact tests and the 95% confidence interval is shown. X-axis is log-scaled, but values shown are before transformation. The right part of the plot denotes the percentage of COPs in each functional annotation.

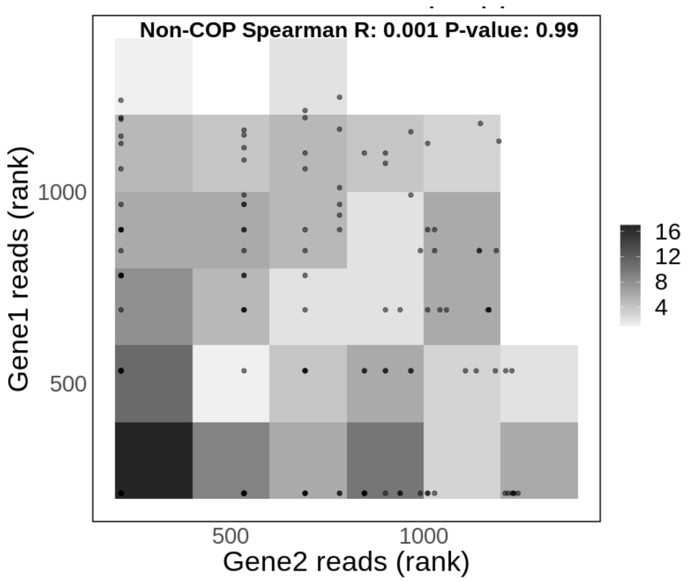

Supplementary Figure 10 GRO-seq read correlation for non-COPs. N = 150. Reads mapping to the TSS positions of each gene were considered. Gene pairs with missing data in at least one of the genes were excluded. The number of reads across all genes in COPs and non-COPs was ranked prior to plotting. Two genes sharing the same number of reads share the same rank.

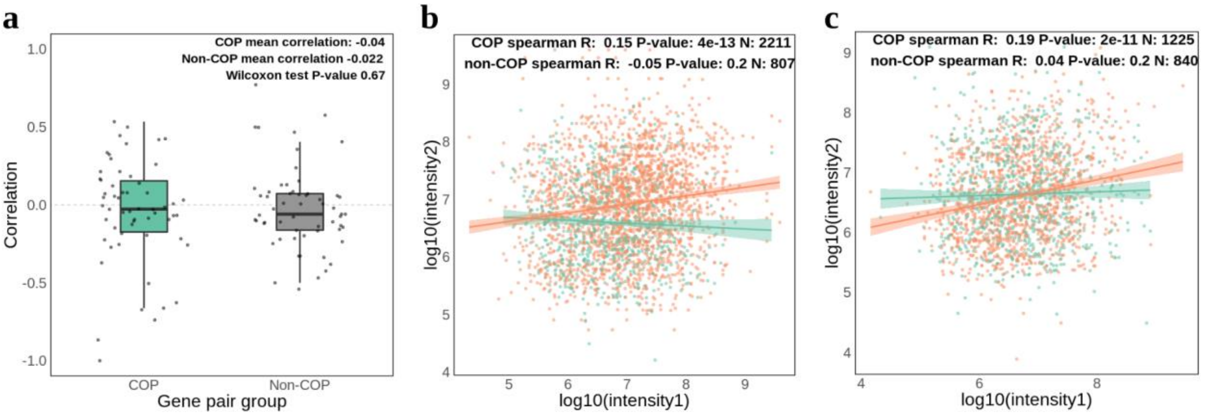

Supplementary Figure 11 Proteomics support for COPs and non-COPs (a) gene pair correlation of protein intensities for each of 58 individual-experiments with proteomics data when shuffling gene pair labels for COPs and non-COPs, separately, on each individual-experiment. Missing data was replaced with 0. The length of the box corresponds to the IQR with the centre line corresponding to the median, the upper and lower whiskers represent the largest or lowest value no further than 1.5 × IQR from the third and first quartile, respectively. **(b)** correlation of protein intensities averaged across 42 individuals for all distinct scCOPs identified in those individuals and their matching non-COPs; **(c)** protein intensity correlation for bulk COPs and non-COPs across all individual-experiments. Fit lines correspond to a linear regression model with 95% confidence intervals.

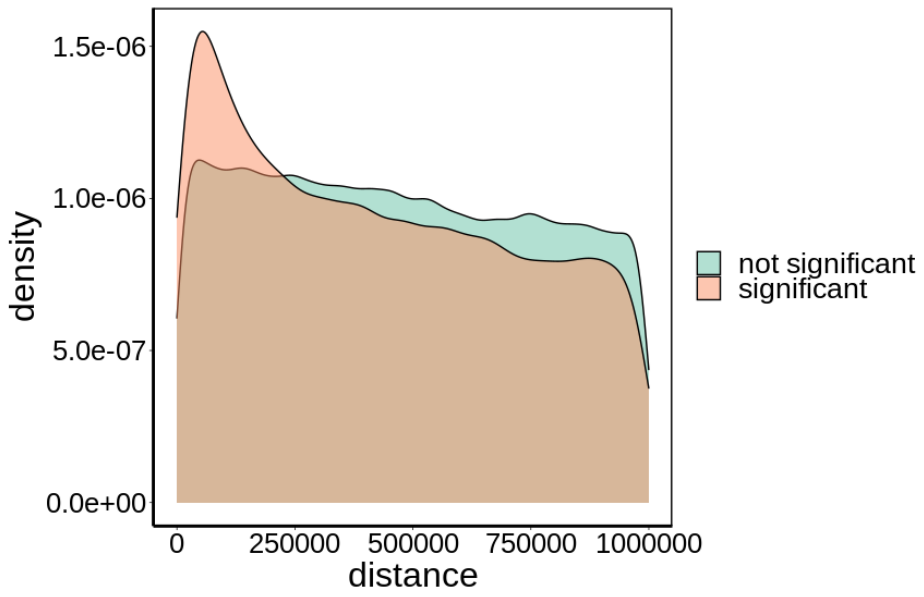

Supplementary Figure 12 Absolute distance distribution between significantly associated and other gene-enhancer pairs tested. The midpoint of the enhancer and the gene TSS were used for the distance calculation. Significant associations were defined as a Spearman correlation > 0.05 and permutation FDR < 5%.

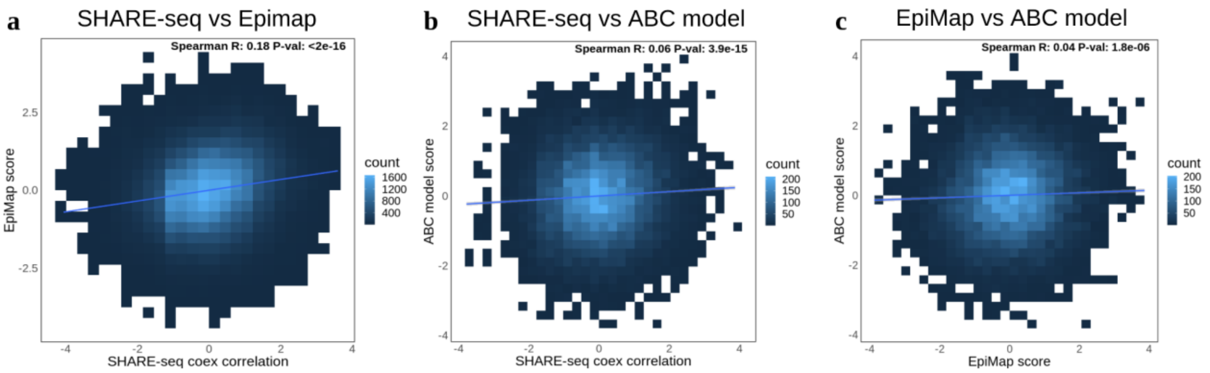

Supplementary Figure 13 Correlation between gene-enhancer association scores between several approaches. (a) SHARE-seq (produced here) versus EpiMap (N = 99,911); **(b)** SHARE-seq versus ABC model (N = 19,024); **(c)** EpiMap versus ABC model (N = 18,375). For each comparison, only gene-enhancer pairs assessed in both associations were considered. Values were quantile normalised (mean = 0, sd = 1). No score or correlation filters were applied.

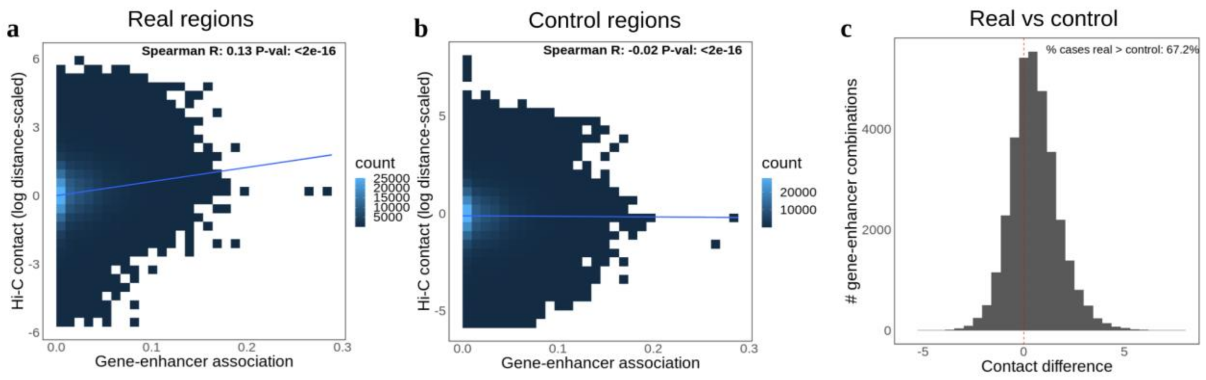

Supplementary Figure 14 Hi-C support of gene-enhancer associations with 5kb resolution. (a) Hi-C contact intensities per gene-enhancer association correlation; **(b)** Hi-C contact intensities per gene-enhancer association correlations when considering control 'enhancer' regions on the opposite up- or down-stream location in respect to the gene TSS; **(c)** Hi-C contact intensity difference between real and control regions. A shift of the distribution to the right (above 0) represents higher Hi-C contacts in the real data compared to control. Missing data (genes or enhancers without Hi-C data) was replaced with 0.

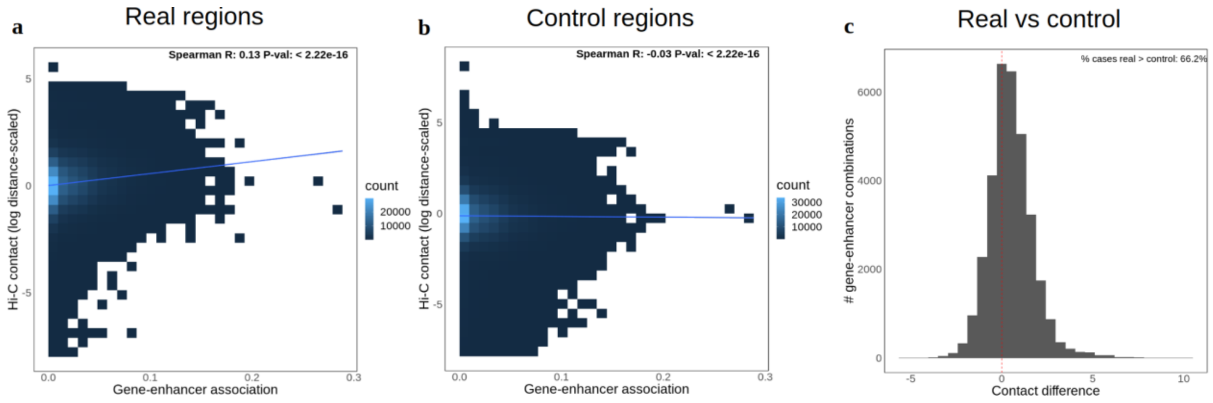

Supplementary Figure 15 Hi-C support of gene-enhancer associations with 10kb resolution. (a) Hi-C contact intensities per gene-enhancer association correlation; **(b)** Hi-C contact intensities per gene-enhancer association correlations when considering control 'enhancer' regions on the opposite up- or down-stream location in respect to the gene TSS; **(c)** Hi-C contact intensity difference between real and control regions. A shift of the distribution to the right (above 0) represents higher Hi-C contacts in the real data compared to control. Missing data (genes or enhancers without Hi-C data) was replaced with 0.

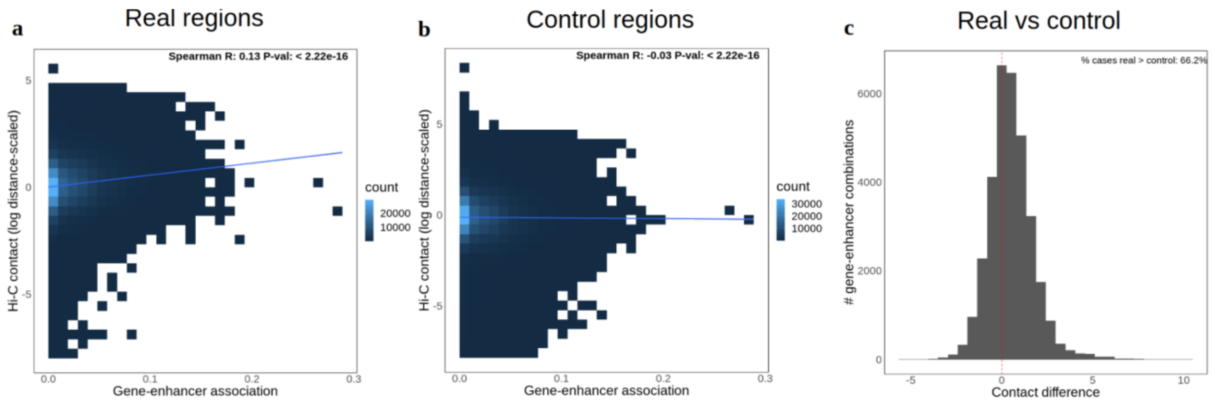

Supplementary Figure 16 Hi-C support of gene-enhancer associations with 25kb resolution. (a) Hi-C contact intensities per gene-enhancer association correlation; **(b)** Hi-C contact intensities per gene-enhancer association correlations when considering control 'enhancer' regions on the opposite up- or down-stream location in respect to the gene TSS; **(c)** Hi-C contact intensity difference between real and control regions. A shift of the distribution to the right (above 0) represents higher Hi-C contacts in the real data compared to control. Missing data (genes or enhancers without Hi-C data) was replaced with 0.

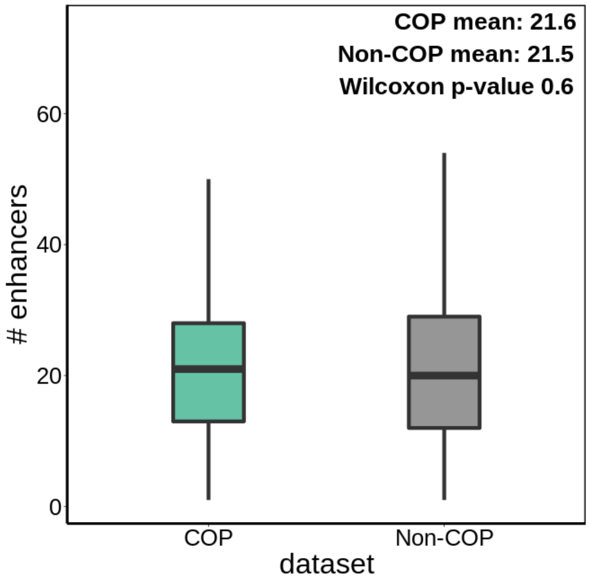

Supplementary Figure 17 Boxplot of the number of enhancers tested per COP and non-COP. The length of the box corresponds to the IQR with the centre line corresponding to the median, the upper and lower whiskers represent the largest or lowest value no further than 1.5 × IQR from the third and first quartile, respectively. Outlier values are not shown.

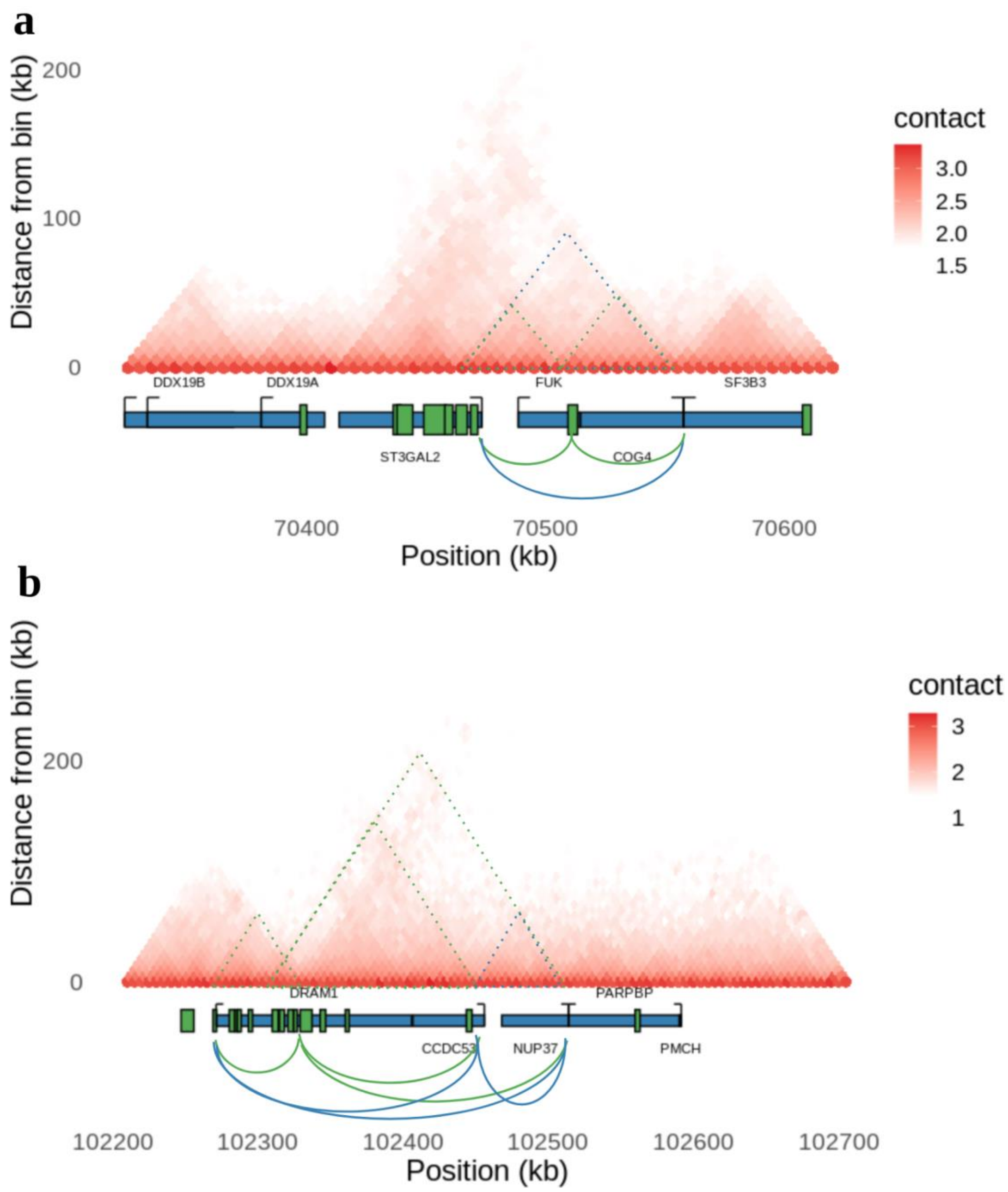

Supplementary Figure 18 Example Hi-C contacts between COPs and shared enhancers (a) region surrounding ST3GAL2 and SF3B3 COP and a shared enhancer (chr16:70510200-70512200); **(b)** region surrounding DRAM1, CCDC53 and NUP37 co-expressed genes, including a region with multiple shared enhancers (within DRAM1 gene model). The blue models depict genes (black dashes represent transcription start site and direction) and blue curves depicts significant gene co-expression (i.e. COP). The green models depict enhancers and green curves depict significant gene-enhancer correlation (correlation > 0.05, FDR < 5%). In addition to the ST3GAL2 and SF3B3 genes, several other Hi-C contacts can be observed between genes and enhancer regions, denoting the complexity of gene regulation architecture.

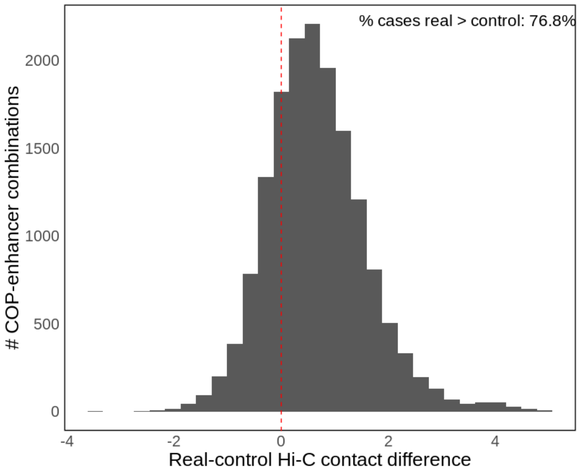

Supplementary Figure 19 Hi-C contact intensity difference between real and control regions for 5kb resolution. Control regions are the opposite up- or down-stream location in respect to the gene TSS. The mean Hi-C contacts between the enhancer and both genes in a COP were used. A shift of the distribution to the right (above 0) represents higher Hi-C contacts in the real data compared to control. Missing data (genes or enhancers without Hi-C data) was replaced with 0.

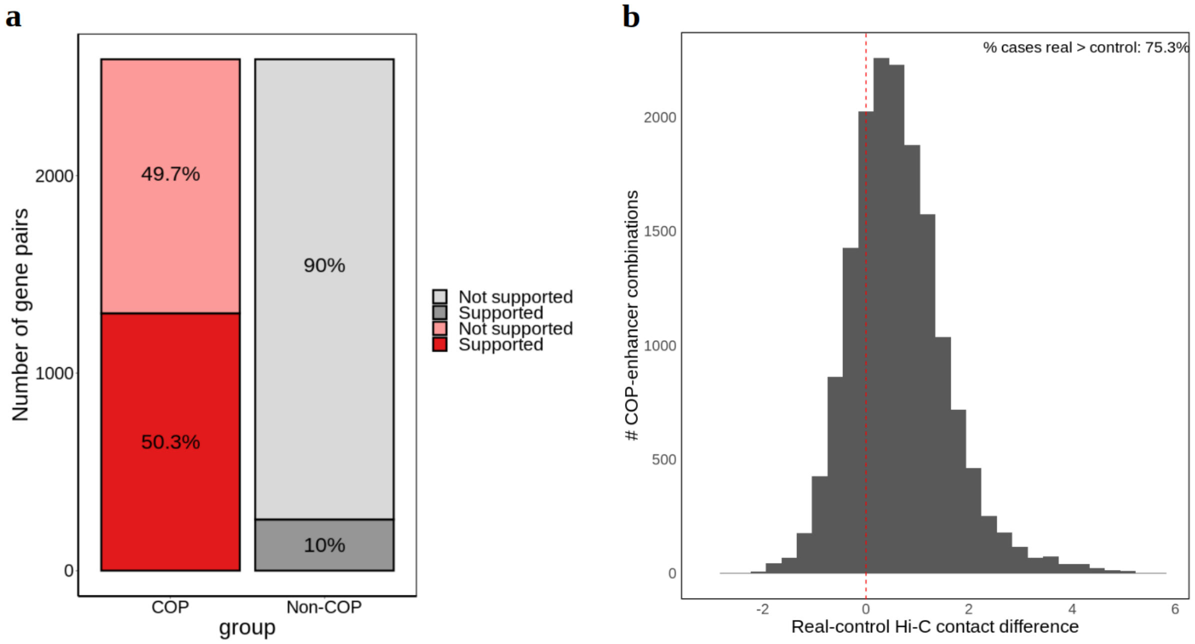

Supplementary Figure 20 Hi-C contact intensity difference between COPs and non-COPs and real and control regions, for 10kb resolution. a number of COPs and non-COPs with Hi-C support (e.g. both enhancer-gene1 and enhancer-gene2 having Hi-C contact higher than the 75th quantile). Note that non-COPs are less likely to share enhancers and thus a smaller number of gene pairs is liable to have Hi-C support. **b** Control regions are the opposite up- or down-stream location in respect to the gene TSS. The mean Hi-C contacts between the enhancer and both genes in a COP were used. A shift of the distribution to the right (above 0) represents higher

Hi-C contacts in the real data compared to control. Missing data (genes or enhancers without Hi-C data) was replaced with 0.

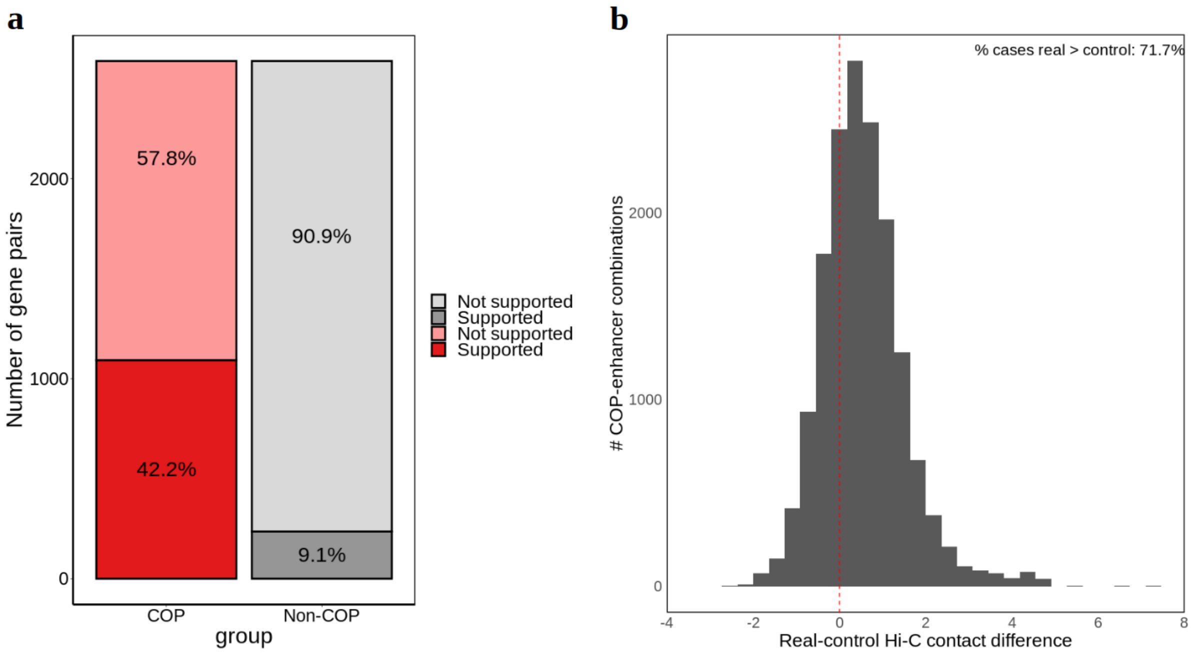

Supplementary Figure 21 Hi-C contact intensity difference between COPs and non-COPs and real and control regions, for 25kb resolution. a number of COPs and non-COPs with Hi-C support (e.g. both enhancer-gene1 and enhancer-gene2 having Hi-C contact higher than the 75th quantile). Note that non-COPs are less likely to share enhancers and thus a smaller number of gene pairs is liable to have Hi-C support. **b** Control regions are the opposite up- or down-stream location in respect to the gene TSS. The mean Hi-C contacts between the enhancer and both genes in a COP were used. A shift of the distribution to the right (above 0) represents higher Hi-C contacts in the real data compared to control. Missing data (genes or enhancers without Hi-C data) was replaced with 0.

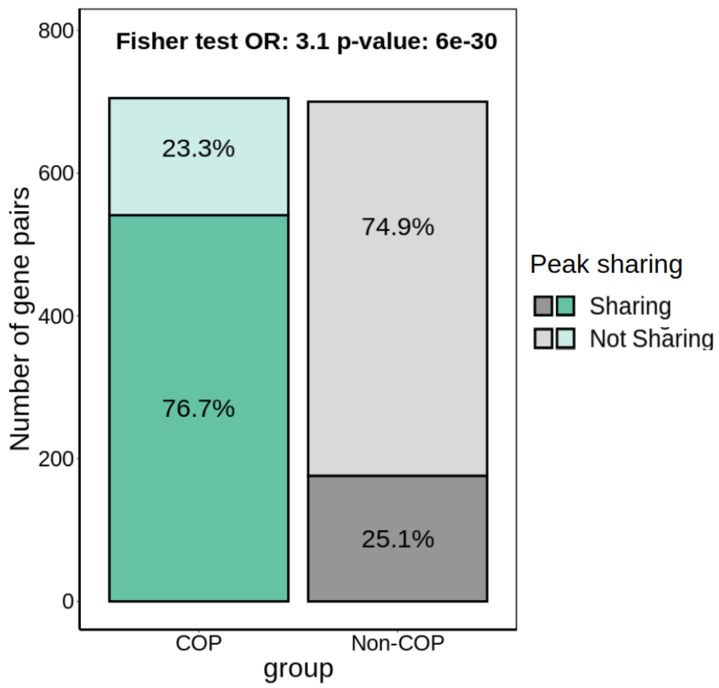

Supplementary Figure 22 Percentage of COPs and non-COPs sharing at least one peak. A total of 31,403 significant gene-peak associations were identified (correlation > 0.05, FDR < 5%).
