# Supplementary material for: Shared regulation and functional relevance of local gene co-expression revealed by single cell analysis
Source: Commun Biol. 2022 Aug 26;5:876. doi: 10.1038/s42003-022-03831-w (PMC9418141; doi:10.1038/s42003-022-03831-w)
Supplement: Supplementary file 3 — Description of Additional Supplementary Files [file 42003_2022_3831_MOESM3_ESM.pdf]

Description of Additional Supplementary Files

File name: Supplementary Data 1
Description: List of single cell co-expressed gene pairs (scCOPs) in iPSC and LCL, as well as bulk COPs in iPSC.

File name: Supplementary Data 2
Description: List of enriched annotations for genes in scCOPs and genes in bulkCOPs.

File name: Supplementary Data 3
Description: Lists of annotations enriched in genes in scCOPs detected from cells in G1, S and G2M cell cycle phases.

File name: Supplementary Data 4
Description: List of 32,883 gene-enhancer associations identified using SHARE-seq LCL data.
