# Supplementary material for: Shared regulation and functional relevance of local gene co-expression revealed by single cell analysis
Source: Commun Biol. 2022 Aug 26;5:876. doi: 10.1038/s42003-022-03831-w (PMC9418141; doi:10.1038/s42003-022-03831-w)
Supplement: Supplementary file 9 — Reporting Summary [file 42003_2022_3831_MOESM9_ESM.pdf]

nature research

Corresponding author(s): Diogo Ribeiro, Olivier Delaneau

Last updated by author(s): Jul 21, 2022

Reporting Summary

Nature Research wishes to improve the reproducibility of the work that we publish. This form provides structure for consistency and transparency in reporting. For further information on Nature Research policies, see our Editorial Policies and the Editorial Policy Checklist.

Statistics

For all statistical analyses, confirm that the following items are present in the figure legend, table legend, main text, or Methods section.

n/a	Confirmed	
☐	☒	The exact sample size (n) for each experimental group/condition, given as a discrete number and unit of measurement
☐	☒	A statement on whether measurements were taken from distinct samples or whether the same sample was measured repeatedly
☐	☒	The statistical test(s) used AND whether they are one- or two-sided<br>*Only common tests should be described solely by name; describe more complex techniques in the Methods section.*
☐	☒	A description of all covariates tested
☐	☒	A description of any assumptions or corrections, such as tests of normality and adjustment for multiple comparisons
☐	☒	A full description of the statistical parameters including central tendency (e.g. means) or other basic estimates (e.g. regression coefficient) AND variation (e.g. standard deviation) or associated estimates of uncertainty (e.g. confidence intervals)
☐	☒	For null hypothesis testing, the test statistic (e.g. F, t, r) with confidence intervals, effect sizes, degrees of freedom and P value noted<br>*Give P values as exact values whenever suitable.*
☒	☐	For Bayesian analysis, information on the choice of priors and Markov chain Monte Carlo settings
☒	☐	For hierarchical and complex designs, identification of the appropriate level for tests and full reporting of outcomes
☐	☒	Estimates of effect sizes (e.g. Cohen's d, Pearson's r), indicating how they were calculated

Our web collection on statistics for biologists contains articles on many of the points above.

Software and code

Policy information about availability of computer code

Data collection	No software was used for the data collection of this study.
Data analysis	All code used in this study is publicly and readily available:<br>Custom scripts: Python v3.6.7 and R v3.4.4<br>BED file manipulation: bedtools v2.29.2<br>Genomic coordinate liftover: USCS liftOver tool (3 Oct 2019, no version)<br>Covariate correction: QTLtools v1.1<br>Gene expression quantification: QTLtools v1.3<br><br>Custom code used for analysis is available under: https://github.com/diogomribeiro/sc_cop

For manuscripts utilizing custom algorithms or software that are central to the research but not yet described in published literature, software must be made available to editors and reviewers. We strongly encourage code deposition in a community repository (e.g. GitHub). See the Nature Research guidelines for submitting code & software for further information.

Data

Policy information about availability of data

All manuscripts must include a data availability statement. This statement should provide the following information, where applicable:

- Accession codes, unique identifiers, or web links for publicly available datasets
- A list of figures that have associated raw data
- A description of any restrictions on data availability

The COPs and enhancer-gene associations produced where are available for download as supplementary tables and through the LoCOP public database (https://

glcoex.unil.ch/). Single cell RNA-seq and ATAC-seq processed data was obtained from the original publications as described in the methods section. Bulk RNA-seq for Yoruba of the 1000 Genomes project were obtained from the EBI ArrayExpress and available through the accession code E-GEUV-1.

Field-specific reporting

Please select the one below that is the best fit for your research. If you are not sure, read the appropriate sections before making your selection.

- [x] Life sciences
- [ ] Behavioural & social sciences
- [ ] Ecological, evolutionary & environmental sciences

For a reference copy of the document with all sections, see nature.com/documents/nr-reporting-summary-flat.pdf

Life sciences study design

All studies must disclose on these points even when the disclosure is negative.

Sample size	Sample sizes were defined by availability of public datasets (not a choice in this study).<br>For the Cuomo et al. 2021 iPSC dataset, 87 European samples with scRNA-seq and bulkRNA-seq were available.<br>For the Sarkar et al. 2019 iPSC dataset, 37 Yoruba samples with scRNA-seq and bulkRNA-seq were available.
Data exclusions	Results from 3 individual-experiment combinations from the Cuomo et al. 2021 dataset and 2 samples from the Sarkar et al. 2019 were excluded on the basis of providing outlier results (number of COPs per number of cells above the 75th quartile + interquartile range (IQR) × 3).
Replication	All the software and data used for this study are publicly available for the replication of the results. Main results were verified in multiple datasets, including the comparison between single cell and bulk RNA-seq results (two datasets were used) and replication of Hi-C contacts across multiple resolutions.
Randomization	No samples were split into experimental groups. When required, analysis considered covariates (PCA-derived).
Blinding	Blinding is not relevant to this study since no group allocation occurs.

Reporting for specific materials, systems and methods

We require information from authors about some types of materials, experimental systems and methods used in many studies. Here, indicate whether each material, system or method listed is relevant to your study. If you are not sure if a list item applies to your research, read the appropriate section before selecting a response.

Materials & experimental systems

n/a	Involved in the study	
☒	☐	Antibodies
☒	☐	Eukaryotic cell lines
☒	☐	Palaeontology and archaeology
☒	☐	Animals and other organisms
☒	☐	Human research participants
☒	☐	Clinical data
☒	☐	Dual use research of concern

Methods

n/a	Involved in the study	
☒	☐	ChIP-seq
☒	☐	Flow cytometry
☒	☐	MRI-based neuroimaging
